# Motivations for participating in a clinical trial on an avian influenza vaccine

**DOI:** 10.1186/1745-6215-13-28

**Published:** 2012-03-27

**Authors:** Laura Costas, José M Bayas, Beatriz Serrano, Sarah Lafuente, M-Amparo Muñoz

**Affiliations:** 1 Adult Vaccination Centre, Preventive Medicine and Epidemiology Service, Hospital Clinic-IDIBAPS, Barcelona, Spain; 2 Unit of Infections and Cancer, Cancer Epidemiology Research Programme, Catalan Institute of Oncology, Barcelona, Spain

**Keywords:** Motivations, Clinical trial, Vaccines, Avian influenza

## Abstract

In this study we describe the sociodemographic characteristics of people participating in a clinical trial on the safety and immunogenicity of a H5N1 influenza vaccine and we identify the main motivations for joining it.

## Letter

### Correspondence/Findings

Recently, the appearance of new influenza strains with the ability of propagating by human- human transmission put both the scientific community and the general public on alert [1,2]. For this reason, the WHO underlines the urgency of developing vaccines to meet the new threat [3]. Knowing the characteristics and motivations of participants in clinical trials may help to improve the recruitment phase of future trials. Here, we describe the sociodemographic characteristics and motivations of people participating in a clinical trial of an influenza vaccine carried out in a university hospital.

The H5N1-008 trial (GlaxoSmithKline) was a multicenter phase III, double-blind, clinical trial conducted to compare the safety and reactogenicity of a monovalent H5N1 influenza vaccine with that of the licensed seasonal influenza vaccine Fluarix™ [4]. All healthy subjects aged ≥ 18 years were eligible for the trial. A total of 5,071 volunteers were recruited from 7 European countries between May and June 2006. Information on local and general symptoms was recorded by each subject using diary cards for the first seven days following each vaccination. Serious adverse events were recorded prospectively, ending at the last visit. The recruitment methods included talks, pamphlets and posters within and outside the hospital, medical faculties, outpatient clinics and other health areas. The study required four visits at the vaccination center of the hospital at days 0, 21, 42 and 180, and a blood extraction at each visit. Participants received two vaccinations at days 0 and 21 (pandemic vaccine or Fluarix™).

A cross-sectional study was carried out, in which all 383 recruits from our center were eligible to participate: on the last visit each participant was provided with a self-administered questionnaire of 12 items with closed answers on socio-demographic data, motivations for participating, how they learned of the trial, and others.

A total of 364 of 383 subjects (95%) answered the questionnaire. Characteristics of the participants are presented in Table 1. When our study population was compared to those who participated in seven European countries, no differences were found regarding age and education [4]. The most-common way of learning about the trial was word of mouth (70.1%; n = 255). Fifty-six (15.4%) subjects had participated in previous studies of vaccines or other drug trials. Eighty-seven percent (n = 318) of participants were willing to enroll in similar studies in the future, and 75.0% of the participants (n = 273) would wish to receive the vaccine if it were to be licensed by the European Medicines Agency and they had been in the control group.

**Table 1:** Basic characteristics of the study population

		*Number*	*Percentage*
**Gender**			
	***Woman***	204	(56.0)
	***Man***	160	(44.0)
**Age **†			
	***18 to 24***	80	(22.0)
	***25 to 44***	161	(44.4)
	***45 to 60***	92	(25.3)
	***> 61***	30	(8.3)
**Education level**			
	***Non-university***	95	(26.0)
	***University***	269	(73.9)
**Profession **†			
	***Health***	138	(38.7)
	***Non-health***	123	(34.5)
	***Inactive***	96	(26.9)
**Knowledge of the study **†			
	***Word of mouth***	255	(70.1)
	***Talks***	35	(9.6)
	***Posters/pamphlets***	33	(9.1)
	***Communication media***	14	(3.8)
	***Others***	18	(4.9)
**Main motivation for participating in the trial**			
	***Collaboration with science***	155	(42.6)
	***Influence of other people***	137	(37.6)
	***Protection against pandemic***	57	(15.7)
	***Coverage of expenses***	15	(4.1)
**Willingness to participate in a similar study in the future**			
	***No***	15	(4.1)
	***Yes***	318	(87.4)
	***DK/DA ***‡	31	(8.5)

† Numbers may not add up to the total because of missing values

‡ Did not know/Did not answer

The most-frequently reported main motivation for joining the study was collaboration with science (n = 155; 42.6%), and it was related to several characteristics of participants (Table 2). It varied non-significantly according to gender (*P *= 0.11), and it was strongly related to their age (*P *< 0.001); participants older than 60 years were more likely to report collaboration with science than the younger age categories, while participants who reported economic reasons were mostly younger than 25 years of age. It also varied with the education level, the profession of participants, and the way in which people learned of the study (*P *= 0.06, 0.01 and < 0.001; respectively). Furthermore, age and the way of learning about the study were strongly related (*P *< 0.001).

**Table 2:** Characteristics of the participants by motivation to participate

	*Main reason to participate*								
		
	Coverage of expenses		Collaboration with science		Protection against pandemic		Influence of other people related with the study		***P*-values **†
	(n = 15)		(n = 155)		(n = 57)		(n = 137)		
	***n***	(%)*	***n***	(%)*	***n***	(%)*	***n***	(%)*	
**Gender**									0.11
***Woman***	8	(3.9)	91	(44.6)	38	(18.6)	67	(32.8)	a) 1.0
									b) 1.0
									c) 0.79
***Man***	7	(4.4)	64	(40.0)	19	(11.9)	70	(43.8)	d) 1.0
									e) 0.51
									f) 0.16
**Age **‡									
***18 to 24***	12	(15.0)	29	(36.3)	10	(12.5)	29	(36.3)	< 0.001
***25 to 44***	3	(1.9)	74	(46.0)	33	(20.5)	51	(31.7)	a) 0.03
									b) 0.13
									c) 0.01
***45 to 60***	0	(0.0)	37	(40.2)	10	(10.9)	45	(48.9)	d) 0.24
									e) 0.37
									f) 0.10
***> 61***	0	(0.0)	15	(50.0)	4	(13.3)	11	(36.7)	
**Education level**									0.06
***Non-university***	3	(3.2)	34	(35.8)	23	(24.2)	35	(36.8)	a) 1.0
									b) 0.91
									c) 1.0
***University***	12	(4.5)	121	(45.0)	34	(12.6)	102	(37.9)	d) 0.05
									e) 1.0
									f) 0.29
**Profession **‡									
***Health***	1	(0.8)	60	(48.8)	19	(15.4)	43	(35.0)	0.01
***Non-health***	4	(2.9)	50	(36.2)	26	(18.8)	58	(42.0)	a) 0.07
									b) 0.27
									c) 0.28
***Inactive***	10	(10.4)	41	(42.7)	12	(12.5)	33	(34.4)	d) 0.85
									e) 0.65
									f) 0.87
**Knowledge of the study **‡									
***Word of mouth***	8	(3.1)	95	(37.3)	36	(14.1)	116	(45.5)	< 0.001
***Talks***	1	(2.9)	21	(60.0)	6	(17.1)	7	(20.0)	a) 1.0
									b) 1.0
									c) 0.04
***Posters/pamphlets***	5	(15.2)	18	(54.5)	6	(18.2)	4	(12.1)	d) 1.0
									e) < 0.01
									f) < 0.01
***Communication media***	0	(0.0)	6	(42.9)	6	(42.9)	2	(14.3)	
***Others***	0	(0.0)	11	(61.1)	3	(16.7)	4	(22.2)	

† Fisher's exact test for homogeneity of proportions between categories. *P*-values labelled from a) to b) correspond to the following multiple comparisons, using Holm correction method: a) Coverage of expenses vs Collaboration with science; b) Coverage of expenses vs Protection against pandemic; c) Coverage of expenses vs Influence of other people; d) Collaboration with science vs Protection against pandemic; e) Collaboration with science vs Influence of other people; f) Protection against pandemic vs Influence of other people. Age, profession and knowledge of the study have been recoded as follows for multiple comparisons: < 45 vs 45+, health vs rest of categories, and word of mouth vs rest of categories, respectively

‡ Numbers may not add up to the total because of missing values

* Row percentages

Motivations for participating in clinical trials can be briefly summarized as follows: possibility of health benefits, obtaining information on the studied disease, willingness to contribute to scientific projects, trusting of specific physicians and economic interests [5-7]. Only 19 men and 38 women in our study reported participating to obtain protection against a possible influenza pandemic. These results are in accordance with a study of an HIV vaccine with healthy people, where only 12% of men and 22% of women agreed strongly that they were motivated by the possible protection against HIV [6]. Although it should be taken cautiously, our results highlight altruistic motives, with 40% of men and 45% of women reporting wanting to collaborate with science, in accordance with the literature [7-10]. Results show that one motivation for participation little explored in previous studies, the influence of other people, was an important factor. Most of the participants in our trial knew somebody related to the study, such as other participants who already had enrolled, and 37.6% reported this motivation for participating. Decisions regarding participation are strongly related to the social network of the subject: the attitudes, knowledge and habits of friends, relatives or colleagues regarding clinical trials influence the decision to join a trial. The small number of participants reporting economic reasons in our study was possibly related to the fact that the amount offered was low (100€), since it was meant to cover travel expenses. It predominantly appealed to younger people for whom the financial incentive was meaningful. Contrary, a review carried out among healthy volunteers found that financial reward was the principal reason for participation reported in 8 of 12 studies [10]. The fact that most of the subjects who chose the economic motivation were younger than 25 years shows an interesting link between the age of participants and their motivations. An open answer for other possible motivations was included in the questionnaire. However, only two people chose this option and in both cases the answer fell within the scope of the four proposed categories. The association between the way of learning about the study and the age of participants suggests that the recruitment method could appeal to people with different characteristics. This fact highlights the importance of taking the above into account while designing trials to avoid biased results.

In conclusion, the main reported motivation for participating was collaboration with science, and it was related to several characteristics of the participants. The strategies involved seemed to appeal to people with different demographics, underscoring the importance of the recruitment design in order to ensure generalizability of results.

## Competing interests

The authors have participated in investigations with GlaxoSmithKline and Sanofi Pasteur MSD vaccines (influenza vaccine and others).

## Authors' contributions

LC and JMB conceived the study, and participated in its design. LC performed the statistical analysis and drafted the manuscript. BS and SL participated in the design of the study. MAM carried out the field work of the study. All authors read and approved the final manuscript.
